# The effect of GM-CSF and predictors of treatment outcome in pediatric septic shock patients

**DOI:** 10.1186/s13052-025-01863-6

**Published:** 2025-02-04

**Authors:** Zhen-Hao Yu, Gui-Xiang Tian, Yao-Dong Wang, Ting-Yan Liu, Peng Shi, Jia-Yun Ying, Wei-Ming Chen, Yu-Feng Zhou, Guo-Ping Lu, Cai-Yan Zhang

**Affiliations:** 1 Department of Critical Care Medicine, Children’s Hospital of Fudan University, Shanghai, China; 2 School of Public Health & Shanghai Institute of Infectious Disease and Biosecurity, Fudan University, Shanghai, China; 3 Pediatric Intensive Care Unit, Anhui Provincial Children’s Hospital, Hefei, China; 4 Clinical Research Unit, Children’s Hospital of Fudan University, Shanghai, China; 5 Institute of Pediatrics, Children’s Hospital of Fudan University, and the Shanghai Key Laboratory of Medical Epigenetics, International Co-laboratory of Medical Epigenetics and Metabolism, Ministry of Science and Technology, Institutes of Biomedical Sciences, Fudan University, Shanghai, China

**Keywords:** Pediatric, Sepstic shock, Immunomodulation, Immunotherapy, GM-CSF

## Abstract

**Background:**

Pediatric septic shock is a critical condition associated with high mortality rates, largely due to sepsis-induced immunosuppression. Granulocyte-macrophage colony-stimulating factor (GM-CSF) has been explored as a therapeutic intervention to counteract this immunosuppression. Despite its potential, the efficacy of GM-CSF in pediatric septic shock has not been clearly established. This study aims to investigate the impact of GM-CSF administration on survival rates and to identify key predictors of treatment outcomes in pediatric septic shock patients.

**Methods:**

We conducted a retrospective cohort study at the Pediatric Intensive Care Unit (PICU) of Children’s Hospital of Fudan University, Shanghai, from January 1, 2019, to December 31, 2023. The study included pediatric patients diagnosed with septic shock, analyzing their demographic data, GM-CSF and adjunctive therapies, laboratory results, and clinical outcomes. We employed univariate and multivariate logistic regression models to assess the influence of GM-CSF on 28-day mortality and identify significant predictors of treatment outcomes.

**Results:**

The study included 200 pediatric patients, with 66 receiving GM-CSF treatment and 134 not treated with GM-CSF. The initial comparison showed a higher 28-day mortality in the GM-CSF group (59.1%) compared to the non-GM-CSF group (35.1%, *P* = 0.001). Notably, after adjustment for confounding factors, multivariate analysis revealed that the effect of GM-CSF treatment on 28-day mortality among pediatric septic shock patients did not reach statistical significance, with an odds ratio (OR) of 0.472 and a 95% confidence interval (CI) ranging from 0.153 to 1.457 (*P* = 0.192). However, the analysis indicated a potential trend suggesting that GM-CSF treatment may contribute to a reduction in 28-day mortality. In addition, significant predictors of treatment outcomes included hematopoietic stem cell transplantation (HSCT), lactic acid (LAC) levels, hospital-acquired septic shock (HASS), red blood cell (RBC) count, and platelet (PLT) count.

**Conclusions:**

GM-CSF treatment may benefit pediatric septic shock patients, especially those with higher lactic acid, and lower RBC and platelet counts. These factors, which are significant predictors of outcomes, should be monitored during therapy.

## Introduction

Sepsis, identified as a life-threatening organ dysfunction resulting from a dysregulated host response to infection [1], poses a critical global health challenge and constitutes the leading cause of mortality in hospitals [2]. In 2017, it was estimated that sepsis afflicted 25 million children worldwide, resulting in over 3 million deaths [3]. The global epidemiological data underline the severity of the problem within pediatric intensive care units (PICU), reporting an incidence rate of pediatric sepsis at 8.2%, coupled with a high mortality rate of 25% [4]. Septic shock, the most severe form of sepsis, is characterized by profound circulatory and cellular metabolic abnormalities, significantly elevating the mortality risk [1]. These pathological changes highlight the complexity and severity of the condition, necessitating urgent and comprehensive medical interventions to enhance patient outcomes.

A significant pathophysiological feature observed in septic shock patients is the interaction between pathogen-induced inflammatory responses and subsequent anti-inflammatory feedback, which often results in severe immunosuppression among a notable subset of these patients [5]. This immunosuppressive state renders patients vulnerable to secondary infections, potentially leading to prolonged immunosuppression, immune system failure, and subsequent physical disabilities, thereby heightening the risk of adverse clinical outcomes [6]. Recent studies indicate the potential reversibility of this immunosuppressed state, sparking interest in immunostimulants as prospective immunotherapeutic interventions. Noteworthy among these are granulocyte-macrophage colony-stimulating factor (GM-CSF), IL-7, IFN-γ, and Thymosin α1 [6–9]. GM-CSF, in particular, is a glycoprotein that plays a pivotal role in the generation of polymorphonuclear neutrophils, monocytes, macrophages, and dendritic cells from hematopoietic progenitor cells within the bone marrow [10]. Therapeutically, GM-CSF is available as a recombinant human protein, which offers promise in boosting the metabolic capacity, function, and proliferation of monocytes [11]. GM-CSF has been explored for its immunomodulatory properties in several clinical trials involving acutely and critically ill patients [12–14].

Despite the clinical interest in GM-CSF for septic shock, research focusing on its efficacy, particularly in pediatric populations, remains sparse and yields inconsistent conclusions. For instance, a randomized controlled trial by Meisel et al. [14] suggested that GM-CSF might reduce the duration of mechanical ventilation and the length of stay in hospitals and intensive care units for septic shock patients. Conversely, another trial by Vacheron et al. [15] reported no significant impact of GM-CSF on 28-day mortality or hospital stay duration in these patients. Notably, existing studies primarily involve adult populations. Given the uncertainties surrounding GM-CSF’s effectiveness in pediatric septic shock, our single-center retrospective study aims to explore the influence of GM-CSF treatment and to identify potential predictors of its therapeutic effect in children with septic shock.

## Methods

### Study design and subjects

This investigation was structured as a five-year, single-center, retrospective cohort study, executed at the Pediatric Intensive Care Unit (PICU) of Children’s Hospital of Fudan University from January 1, 2019, to December 31, 2023. Participants were included based on the following criteria: (1) age range between 29 days and 18 years; (2) diagnosis of pediatric septic shock as per the 2015 Chinese Pediatric Expert Consensus, which includes: hypotension (blood pressure below the 5th percentile for age, or systolic blood pressure more than two standard deviations below the normal for age), necessity for vasoactive medications (dopamine > 5 µg/kg/min, or any dose of dobutamine, norepinephrine, or epinephrine), and at least three indicators of hypoperfusion such as weakened peripheral arterial pulsations, skin pallor or mottling, prolonged capillary refill time (> 3 s, excluding environmental influences), early irritability or lethargy progressing to confusion or coma, reduced urine output (< 0.5 mL/kg/h for over 2 h following fluid resuscitation), or lactic acidosis (arterial blood lactate > 2 mmol/L) [16]. Exclusion criteria comprised incomplete medical records.In the PICU, GM-CSF treatment was administered when a patient’s absolute neutrophil count fell below 0.5 × 10^9/L. The study was approved by the Ethics Committee of the Children’s Hospital of Fudan University [No.97 (2024)]. Written informed consent was obtained from all participants or their legal guardians.

### Groups

Patients were retrospectively divided into two groups: those who received GM-CSF treatment (GM-CSF group) and those who did not (No GM-CSF group).

### Data collection

Clinical and demographic data were systematically extracted from the electronic medical records for this study. The collected information included age, sex, initial diagnosis, and detailed laboratory test results. Additionally, data on the administered treatments were recorded, including antibiotic therapy, blood transfusions, use of vasopressors or steroids, mechanical ventilation, and renal replacement therapy. The origin of admission (either from the emergency department or hospital ward) and the specific reasons for admission to the PICU were also documented. The severity of illness upon admission was assessed using the Pediatric Critical Illness Score (PCIS) [17]. The primary study outcomes were defined as 28-day mortality, in-hospital mortality, and the duration of PICU hospitalization. These metrics were used to evaluate the effectiveness of therapeutic interventions and to understand the prognostic factors in pediatric septic shock.

### Definitions

The primary outcome was 28-day mortality, defined as death within 28 days following a diagnosis of septic shock [16]. Septic shock was classified as either community-acquired septic shock (CASS) or hospital-acquired septic shock (HASS) based on the timing of infection onset relative to hospital admission: CASS was defined as septic shock occurring within 48 h of admission, and HASS as occurring after 48 h, inclusive of this time frame [16]. Underlying diseases were considered those pre-existing conditions present prior to the onset of septic shock but not directly contributing to it.

### Statistical analysis

Statistical assessments were conducted using IBM SPSS Statistics 26. The normality of continuous variables was tested with the Kolmogorov–Smirnov test. Continuous data following a normal distribution were compared using the Student’s t-test, whereas those not normally distributed were analyzed using the Mann-Whitney U test. Categorical data were evaluated using Pearson’s chi-square test. Normally distributed continuous variables were expressed as mean ± standard deviation; non-normally distributed data were presented as median and interquartile range. Categorical variables were reported as frequency and percentage. Both univariate and multivariable logistic regression analyses were employed to explore the potential predictors of treatment outcomes with GM-CSF in septic shock patients. The Receiver Operating Characteristic (ROC) curve analysis determined cutoff values for categorization, with certain variables like white blood cell (WBC) count segmented into quartiles. Results were expressed as adjusted odds ratios (ORs) with 95% confidence intervals (CIs). A p-value of less than 0.05 was considered statistically significant.

## Results

### Demographic characteristics of patients

**Fig. 1:**
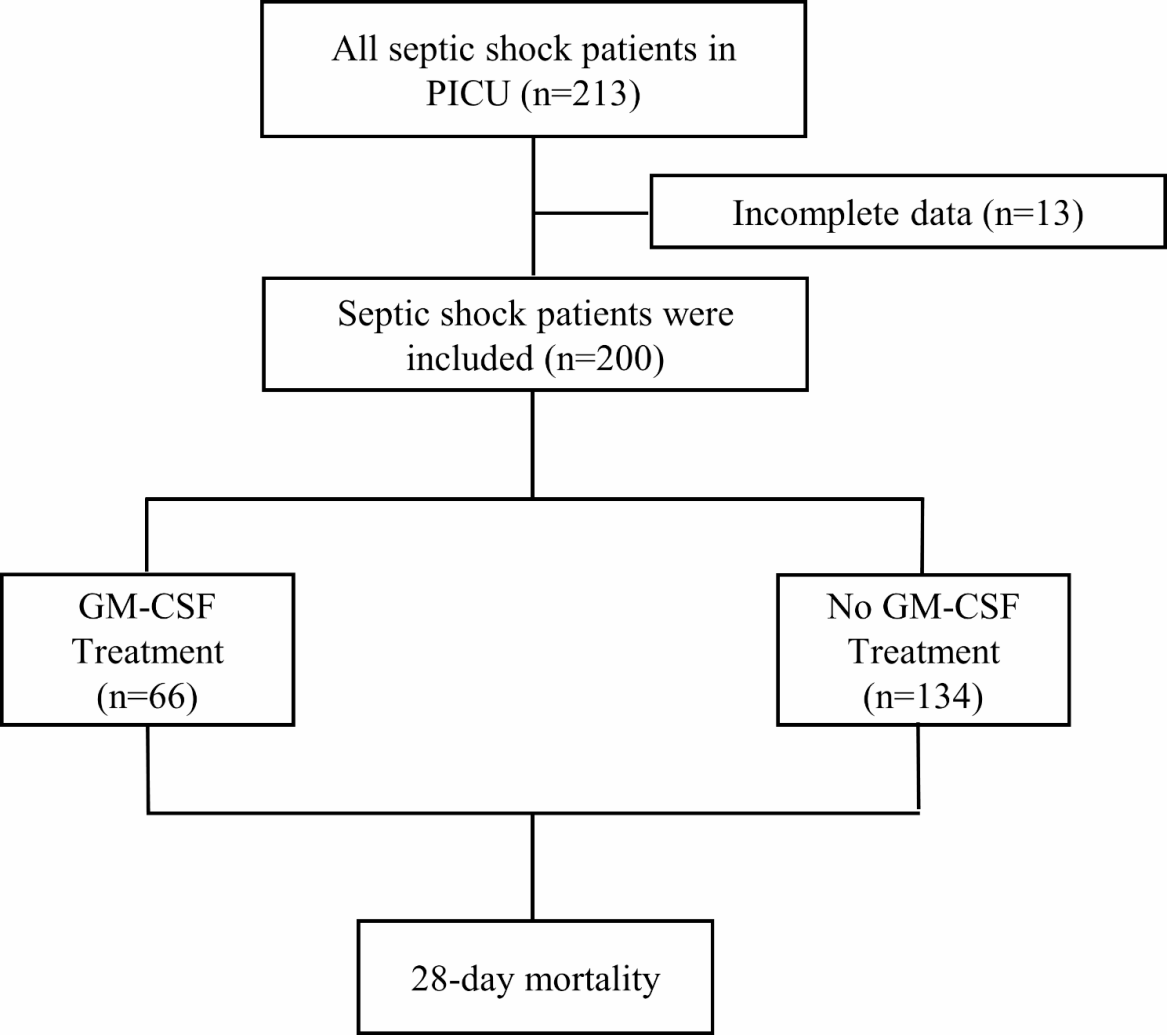
Flowchart of the retrospective cohort study

Over a five-year period, the study enrolled 213 pediatric patients diagnosed with septic shock, though 13 were excluded due to incomplete data (Fig. 1). Of the remaining 200 patients, 66 (33%) were treated with GM-CSF and 134 (67%) were not. The GM-CSF group predominantly consisted of patients with pre-existing conditions (98.5%), notably hematologic or oncologic disorders (66.7%), and hematopoietic stem cell transplantation (HSCT) recipients (27.3%). The median age in the GM-CSF cohort was 6.8 years (interquartile range: 2.4 to 11.8), and 66.7% were male. Detailed demographic characteristics and baseline laboratory values, which differed significantly between the two groups, are presented in Table 1.

**Table 1:** Study cohort characteristics and laboratory indicators at septic shock diagnosis

Characteristics	GM-CSF (*n* = 66)	No GM-CSF (*n* = 134)	*P*
Age, year	6.8 (2.4, 11.8)	3.5 (0.8, 8.9)	0.001
Male, n (%)	44 (66.7)	85 (63.4)	0.653
Underlying diseases, n (%)			
≥1 obvious underlying disease	65 (98.5)	95 (70.9)	< 0.001
Hematologic/oncologic diseases	44 (66.7)	20 (14.9)	< 0.001
Gene deficiency	17 (25.8)	34 (25.4)	0.953
Autoimmune disease	4 (6.1)	3 (2.2)	0.330
HSCT	18 (27.3)	12 (9.0)	0.001
Nervous system disease	0 (0.0)	15 (11.2)	0.011
Digestive tract disease	0 (0.0)	9 (6.7)	0.073
Congenital heart disease	0 (0.0)	3 (2.2)	0.544
ICU admission source, n (%)			< 0.001
Emergency	18 (27.3)	79 (59.0)	
Hospital Ward	48 (72.7)	55 (41.0)	
Reason for ICU admission, n (%)			0.311
Septic shock	27 (40.9)	65 (48.5)	
Other	39 (59.1)	69 (51.5)	
PCIS at SS diagnosis, median (IQR)	72.0 (68.0, 80.0)	75.0 (60.0, 80.0)	0.208
Vital signs at SS diagnosis			
Temperature (℃), median (IQR)	37.2 (36.5, 38.3)	37.0 (36.5, 37.8)	0.375
Abnormal RR, n (%)	28 (42.4)	57 (42.5)	0.988
Abnormal HR, n (%)	38 (57.6)	76 (56.7)	0.908
Source of infection, n (%)			< 0.001
HASS	45 (68.2)	35 (26.1)	
CASS	21 (31.8)	99 (73.9)	
Positive pathogen detection, n (%)	37 (56.1)	77 (57.5)	0.851
G+	7 (10.6)	23 (17.2)	0.222
G-	24 (36.4)	48 (35.8)	0.940
Virus	5 (7.6)	17 (12.7)	0.398
Fungus	12 (18.2)	11 (8.2)	0.038
Indicators, Median (IQR)			
RBC (×10^9^/L)	2.34 (2.05, 2.97)	3.45 (2.69, 4.05)	< 0.001
Hb (g/L), mean (SD)	72.7 (19.8)	93.8 (27.8)	< 0.001
WBC (×10^9^/L)	0.59 (0.19, 1.41)	9.70 (5.64, 15.00)	< 0.001
PLT (×10^9^/L)	17 (5, 32)	135 (75, 213)	< 0.001
N (×10^9^/L)	0.00 (0.00, 0.57)	7.13 (3.83, 13.05)	< 0.001
M (×10^9^/L)	0.00 (0.00, 0.10)	0.56 (0.22, 1.29)	< 0.001
L (×10^9^/L)	0.00 (0.00, 0.47)	1.40 (0.60, 3.00)	< 0.001
CRP (mg/L)	119.0 (23.0, 141.8)	47.9 (12.0, 125.0)	0.012
LAC (mmol/L)	4.7 (2.1, 9.2)	2.9 (1.5, 5.3)	0.503
PH	7.33 (7.06, 7.46)	7.39 (7.26, 7.49)	0.471
GLU (mmol/L)	7.7 (6.4, 13.9)	8.9 (6.8, 12.3)	0.848
Na (mmol/L)	136 (132, 140)	135 (131, 140)	0.253
K (mmol/L)	3.4 (2.9, 4.2)	3.1 (2.6, 5.1)	0.445
INR	1.45 (1.24, 1.78)	1.50 (1.29, 1.94)	0.164
PT (s)	17.8 (15.9, 20.2)	18.3 (16.1, 22.5)	0.180
APTT (s)	41.9 (38.2, 49.8)	40.4 (34.2, 55.6)	0.746
d-Dimer (mg/L)	1.84 (1.14, 7.43)	3.40 (1.62, 7.19)	0.047
ALB (g/L)	28.2 (24.5, 31.9)	29.8 (26.9, 33.1)	0.037
TBIL (µmol/L)	28.5 (12.5, 100.1)	12.5 (6.7, 44.7)	0.014
ALT (U/L)	38.4 (14.6, 114.2)	37.6 (14.5, 129.2)	0.242
AST (U/L)	108.5 (36.2, 280.6)	77.8 (37.4, 348.6)	0.031
CR (µmol/L)	30.8 (24.0, 56.0)	43.8 (24.0, 84.0)	0.045
Urea (mmol/L)	6.08 (4.75, 8.51)	7.00 (3.81, 10.55)	0.742

*HSCT*, Hematopoietic stem cell transplant; *GM-CSF*, Granulocyte-macrophage colony-stimulating factor; *PCIS*, Pediatric critical illness score; *SS*, Septic shock; *RR*, Respiratory rate; *HR*, Heart rate; *HASS*, Hospital-acquired septic shock; *CASS*, Community-acquired septic shock; *RBC*, Red blood cell; *HB*, Hemoglobin; *WBC*, White blood cell; *PLT*, Platelet; *N*, Neutrophil; *M*, Monocyte; *L*, Lymphocyte; *CRP*, C-reactive protein; *LAC*, Lactic acid concentration; *Na*, Sodium; *K*, Potassium; *INR*, International normalized ratio; *PT*, Prothrombin time; *APTT*, Activated partial thromboplastin time; *ALB*, Albumin; *TBIL*, Total bilirubin; *ALT*, Alanine aminotransferase; *AST*, Aspartate aminotransferase; *CR*, Creatinine

### Supportive therapies

Our analysis found no significant differences in the overall use of supportive therapies between the groups. These therapies included initial empirical antibacterial treatment, respiratory support, renal replacement therapy, and steroid administration. However, the use of norepinephrine was significantly higher in the GM-CSF group (98.5%) compared to those not receiving GM-CSF (85.8%; *P* = 0.005), as shown in Table 2. No other differences in the use of vasoactive agents were observed.

**Table 2:** Antimicrobial and supportive therapies in pediatric patients with septic shock

Characteristics	GM-CSF (*n* = 66)	No GM-CSF (*n* = 134)	*P*
Types of empirical antimicrobial therapy on day 1, n (%)			
Carbapenems	48 (72.7)	95 (70.9)	0.787
Glycopeptides	30 (45.5)	64 (47.8)	0.759
Oxazolidinone	12 (18.2)	21 (15.7)	0.653
Beta-lactamase inhibitors	1 (1.5)	4 (3.0)	0.885
Cephalosporin	13 (19.7)	17 (12.7)	0.192
Aminoglycosides	3 (4.5)	4 (3.0)	0.876
Quinolones	9 (13.6)	13 (9.7)	0.403
Nitroimidazoles	0 (0.0)	7 (5.2)	0.139
Sulfanilamide	13 (19.7)	17 (12.7)	0.192
Glycyl tetracyclines	6 (9.1)	6 (4.5)	0.196
Macrolide antibiotics	1 (1.5)	4 (3.0)	0.885
Polypeptide antibiotic	8 (12.1)	5 (3.7)	0.050
Antiviral drugs, n (%)	18 (27.3)	20 (14.9)	0.036
Antifungal drugs, n (%)	32 (48.5)	36 (26.7)	0.002
Respiratory support	65 (98.5)	133 (93.3)	0.607
Ventilator days, median (IQR)	13.0 (7.0, 22.0)	13.0 (6.0, 36.0)	0.543
Renal replacement therapy, n (%)	23 (34.8)	59 (44.0)	0.214
Transfusions, n (%)	22 (33.3)	57 (42.5)	0.211
Vasoactive agents, n (%)			
Norepinephrine	65 (98.5)	115 (85.8)	0.005
Epinephrine	42 (63.6)	66 (49.3)	0.055
Dobutamine	60 (90.9)	108 (80.6)	0.061
Terlipressin	9 (13.6)	17 (12.7)	0.851
Milrinone	12 (18.2)	13 (9.7)	0.088
Vasopressor days, median (IQR)	5.0 (4.0, 8.0)	5.0 (3.0, 8.0)	0.636
Steroids, n (%)	39 (59.1)	62 (46.3)	0.088

*GM-CSF*, Granulocyte-macrophage colony-stimulating factor

### Clinical outcomes and complications

Patients in the GM-CSF group exhibited a higher incidence of disseminated intravascular coagulation (DIC; 45.5% vs. 24.6%, *P* = 0.003). Although there were no significant differences in the occurrence of respiratory failure or other complications, the analysis revealed a significantly higher 28-day mortality rate (59.1% vs. 35.1%, *P* = 0.001) and in-hospital mortality rate (43.9% vs. 26.1%, *P* = 0.011) in the GM-CSF group compared to those who did not receive GM-CSF. Nevertheless, no statistically significant difference was found in the duration of ICU stay (*P* = 0.351) (Table 3).

**Table 3:** Complications and outcomes in pediatric patients with septic shock

Characteristics	GM-CSF (*n* = 66)	No GM-CSF (*n* = 134)	*P*
Prognosis			
Length of ICU stay, median (IQR)	13.5 (8.0, 26.0)	15.0 (8.0, 36.0)	0.351
28-day mortality, n (%)	39 (59.1)	47 (35.1)	0.001
In-hospital mortality, n (%)	29 (43.9)	35 (26.1)	0.011
Complications, n (%)			
Respiratory failure	49 (74.2)	106 (79.1)	0.439
Acute renal failure	16 (24.2)	26 (19.4)	0.429
Liver dysfunction	10 (15.2)	26 (19.4)	0.462
DIC	30 (45.5)	33 (24.6)	0.003
Cerebral dysfunction	9 (13.6)	30 (22.4)	0.142
Digestive failure	11 (16.7)	14 (10.4)	0.211
MODS	53 (80.3)	117 (87.3)	0.192

*GM-CSF*, Granulocyte-macrophage colony-stimulating factor; *DIC*, Disseminated intravascular coagulation; *MODS*, Multiple organ dysfunction syndrome

### Potential predictive factors

Initial analyses using univariate logistic regression indicated GM-CSF as a risk factor for 28-day mortality (OR 2.674; CI 1.459–4.899, *P* = 0.001). However, subsequent multivariable analysis, which adjusted for demographic characteristics, disease severity, and laboratory markers, suggested a potential protective effect of GM-CSF on 28-day mortality, although this finding was not statistically significant (OR 0.472; CI 0.153–1.457, *P* = 0.192). Further regression modeling identified several important predictors related to the effects of GM-CSF on 28-day mortality. These included HSCT (*P* = 0.024), high LAC (*P* < 0.001), HASS (*P* = 0.018), low RBC (*P* = 0.022), and low PLT (*P* < 0.001) as detailed in Table 4.

**Table 4:** Univariate and multivariate logistic regression analysis of 28-day mortality in children with septic shock

Variable	Univariate logistic regression			Multivariate logistic regression							
	OR (95% CI)	*P*		Model1			Model2			Model3	
				OR (95% CI)	*P*		OR (95% CI)	*P*		OR (95% CI)	*P*
Age, year	1.003 (0.947–1.064)	0.907		1.028 (0.953–1.109)	0.471		1.061 (0.975–1.155)	0.168		1.059 (0.971–1.155)	0.197
Sex	1.048 (0.584–1.883)	0.874		0.965 (0.484–1.925)	0.919		1.043 (0.494–2.204)	0.912		1.022 (0.474–2.205)	0.956
GM-CSF	2.674 (1.459–4.899)	**0.001**		1.278 (0.501–3.259)	0.608		0.515 (0.196–1.358)	0.180		0.472 (0.153–1.457)	0.192
HSCT	4.690 (2.055–10.704)	**< 0.001**		3.851 (1.458–10.170)	**0.006**		3.799 (1.342–10.751)	**0.012**		3.569 (1.183–10.767)	**0.024**
PCIS											
> 80	Ref.			Ref.			Ref.			Ref.	
71–80	2.277 (1.039–4.989)	**0.040**		1.947 (0.737–5.140)	0.179		2.259 (0.792–6.444)	0.127		2.018 (0.694–5.874)	0.198
≤ 70	2.110 (0.964–4.618)	0.062		1.325 (0.491–3.580)	0.578		1.399 (0.487–4.015)	0.533		1.208 (0.395–3.693)	0.740
LAC, mmol/L											
≤ 2.3	Ref.			Ref.			Ref.			Ref.	
> 2.3	4.849 (2.581–9.110)	**< 0.001**		4.594 (2.256–9.357)	**< 0.001**		4.360 (2.049–9.277)	**< 0.001**		4.457 (2.046–9.708)	**< 0.001**
WBC, 10^9^/L											
P_25_-P_75_(0.81–13.42)	Ref.			Ref.			-			Ref.	
< P_25_ (0.81)	2.160 (1.083–4.039)	**0.029**		1.214 (0.465–3.171)	0.692		-			0.671 (0.229–1.968)	0.468
> P_75_ (13.42)	0.880 (0.435–1.778)	0.721		1.234 (0.546–2.790)	0.614		-			1.663 (0.667–4.145)	0.275
HASS vs. CASS	3.546 (1.958–6.420)	**< 0.001**		2.909 (1.382–6.125)	**0.005**		-	-		2.728 (1.189–6.259)	**0.018**
CRP, mg/L											
≤ 8	Ref.			Ref.			-	-		Ref.	
> 8	0.940 (0.245–3.612)	0.929		1.385 (0.510–3.762)	0.523		-	-		1.046 (0.339–3.233)	0.937
RBC, 10^9^/L											
≤ 2.80	3.942 (2.177–7.139)	**< 0.001**		-	-		2.535 (1.158–5.549)	**0.020**		2.571 (1.147–5.761)	**0.022**
> 2.80	Ref.			-	-		Ref.			Ref.	
PLT, 10^9^/L											
≤ 74	7.765 (4.089–14.745)	**< 0.001**		-	-		6.725 (2.844–15.902)	**< 0.001**		7.369 (2.947–18.523)	**< 0.001**
> 74	Ref.			-	-		Ref.			Ref.	

*GM-CSF*, Granulocyte-macrophage colony stimulating factor; *HSCT*, Hematopoietic stem cell transplant; *PCIS*, Pediatric critical illness score; *LAC*, Lactic acid concentration; *WBC*, White blood cell; *HASS*, Hospital-acquired septic shock; *CASS*, Community-acquired septic shock; *CRP*, C-reactive protein; *RBC*, Red blood cell; *PLT*, Platelet

## Discussion

In this retrospective, single-center cohort study, which included 200 patients diagnosed with septic shock, our findings suggest that GM-CSF might serve as a potential protective factor that could contributes to a reduction in 28-day mortality, as determined through our multivariable regression analysis. Furthermore, our study identified critical predictors of treatment outcomes, notably elevated lactate levels, reduced RBC, and lower PLT, which collectively contribute to our understanding of the prognostic factors in pediatric septic shock management.

This retrospective study explored the associations between several predictive variables and the outcomes of pediatric septic shock, with a specific focus on 28-day mortality as the dependent variable and various factors, including GM-CSF treatment, patients’ baseline conditions, infection status, and hematologic indicators, as independent variables. GM-CSF, a pro-inflammatory cytokine, is known for its role in stimulating bone marrow to produce granulocytes and macrophages, which helps counteract immune suppression observed in septic shock patients [18]. However, our univariate logistic regression analysis revealed a higher 28-day mortality rate among patients receiving GM-CSF therapy. To address the baseline differences among patients regarding GM-CSF treatment, we utilized a multivariable regression model. The final OR for GM-CSF treatment was 0.472 (95% CI: 0.153–1.457), suggesting a possible protective effect, though this result did not reach statistical significance. This finding contrasts with results from randomized controlled trials by Meisel [14] and Vacheron [15], which showed that GM-CSF treatment had minimal effect on patients’ 28-day mortality. Several factors may explain these discrepancies. First, there is a variation in the study populations. Prior studies predominantly involved adult patients and focused on individuals with abnormal monocyte human leukocyte antigen-DR (mHLA-DR) levels, whereas our study encompasses a broader spectrum of immunosuppressed pediatric patients. Second, the methodological differences between our retrospective cohort study and the randomized controlled trials conducted by Meisel and Vacheron likely contribute to the divergent outcomes observed in the 28-day mortality rates. These findings underscore the complexity of treating pediatric septic shock and suggest that while GM-CSF has potential benefits, its efficacy may vary depending on patient-specific factors and study methodologies.

In our analysis, we observed a significant difference in the effects of GM-CSF treatment on patient outcomes between univariate and multivariate models. This prompted further investigation into the key predictors associated with outcomes in our model, such as HSCT, LAC, HASS, RBC, and low PLT, all of which may influence 28-day mortality. The principal predictors impacting the efficacy of GM-CSF treatment included baseline patient conditions, specifically HSCT and elevated lactate levels. Our study identified two primary groups significantly associated with GM-CSF use in septic shock: patients with a history of tumor chemotherapy and those undergoing HSCT. While the use of GM-CSF as adjunctive therapy aims to enhance immune function in neutropenic patients [19–21], only HSCT emerged as a significant predictor. Patients undergoing HSCT typically exhibit poorer baseline conditions and are more susceptible to various mortality risk factors due to graft-versus-host disease and infections [22]. Elevated lactate levels are also a critical predictor, indicating tissue hypoxia and potentially exacerbating the suppressive effects on immune cells, which compromises the body’s immune response capabilities [23, 24]. A retrospective cohort study involving 1,060 patients diagnosed with septic shock demonstrated a significant correlation between lactate levels at 6 h post-onset and 28-day mortality (OR with 95% CI, 1.27 [1.21–1.34]) [25]. This finding underscores the relevance of high lactate levels as a predictor of adverse outcomes. Lastly, among infection-related factors, HASS was identified as the most significant predictor. Previous research have consistently highlighted a higher 28-day mortality among HASS patients compared to those with CASS [16, 26], underscoring the importance of considering HASS as a predictor of outcomes. Further exploration of these associations is essential to understand the complex interplay between treatment strategies and patient-specific variables in pediatric septic shock.

Our study underscores the critical role of hematologic status, particularly RBC and PLT counts, in predicting outcomes in pediatric septic shock patients. RBCs are fundamental not only for oxygen and carbon dioxide transport and exchange but also play vital roles in cellular blood immunity through mechanisms such as chemokine regulation, complement binding, and pathogen immobilization [27, 28]. In sepsis, reduced RBC counts may result from a variety of factors including functional iron deficiency, ongoing infection, inflammation, or multiple organ dysfunction syndrome [29]. While RBC count alone may not be a standalone diagnostic or prognostic marker for sepsis, it serves as a valuable indicator of the severity of the disease [30]. In our retrospective analysis, patients with septic shock who presented with lower RBC levels frequently experienced poorer clinical outcomes, highlighting the importance of monitoring hematologic variables in this patient population. Similarly, platelets play a crucial role in hemostasis and significantly modulate inflammatory responses [31]. In the context of sepsis and septic shock, thrombocytopenia is commonly observed and is generally a result of both diminished production and increased consumption of platelets [32]. Consistent with the literature, our findings indicate that lower platelet counts are strongly associated with increased mortality in intensive care settings [33, 34]. This is corroborated by a prospective study of 931 patients with septic shock, where those with lower and intermediate-low platelet counts exhibited significantly increased risks of 30-day mortality compared to those with normal platelet levels (hazard ratios with 95% CI, 2.00 [1.32–3.05] and 1.72 [1.22–2.44]), respectively [35].

Despite these insights, the study has several limitations. As an observational study, the results need validation through larger-scale, randomized controlled trials (RCTs). Being a single-center study, the findings may have limited generalizability. Furthermore, a longer follow-up period is necessary to assess long-term clinical outcomes adequately.

## Conclusion

This study elucidates the clinical advantages of GM-CSF treatment in pediatric patients with septic shock, highlighting the prognostic significance of higher lactate levels, and lower RBC and PLT counts. These findings underscore the necessity of closely monitoring these parameters during treatment to optimize clinical outcomes. Although our retrospective cohort study involved a substantial number of children with septic shock, the results warrant confirmation through further double-blind, placebo-controlled trials. Moving forward, our research aims to identify specific biomarkers that can predict which pediatric septic shock patients will derive the most benefit from GM-CSF therapy, thereby refining therapeutic approaches and enhancing patient outcomes. This effort aligns with the broader goal of advancing personalized medicine in pediatric critical care.

## Data Availability

The datasets generated and analyzed during the current study are available from the corresponding author on reasonable requests.
